# The Current State of Mobile Phone Apps for Monitoring Heart Rate, Heart Rate Variability, and Atrial Fibrillation: Narrative Review

**DOI:** 10.2196/11606

**Published:** 2019-02-15

**Authors:** Ka Hou Christien Li, Francesca Anne White, Timothy Tipoe, Tong Liu, Martin CS Wong, Aaron Jesuthasan, Adrian Baranchuk, Gary Tse, Bryan P Yan

**Affiliations:** 1 Department of Medicine and Therapeutics Faculty of Medicine The Chinese University of Hong Kong Hong Kong China (Hong Kong); 2 Li Ka Shing Institute of Health Sciences Faculty of Medicine The Chinese University of Hong Kong Hong Kong China (Hong Kong); 3 Faculty of Medicine Newcastle University Newcastle upon Tyne United Kingdom; 4 Tianjin Key Laboratory of Ionic-Molecular Function of Cardiovascular Disease Department of Cardiology, Tianjin Institute of Cardiology Second Hospital of Tianjin Medical University Tianjin China; 5 Division of Family Medicine and Primary Health Care JC School of Public Health and Primary Care, Faculty of Medicine The Chinese University of Hong Kong Hong Kong China (Hong Kong); 6 Division of Cardiology Kingston General Hospital Queen's University Kington, ON Canada; 7 Division of Cardiology Department of Medicine and Therapeutics, Faculty of Medicine The Chinese University of Hong Kong Hong Kong China (Hong Kong); 8 Division of Cardiology Department of Medicine and Therapeutics Prince of Wales Hospital Hong Kong China (Hong Kong); 9 Institute of Vascular Medicine The Chinese University of Hong Kong Hong Kong China (Hong Kong)

**Keywords:** mobile phone apps, atrial fibrillation, heart rate, arrhythmia, photoplethysmography, electrocardiography, mobile health

## Abstract

**Background:**

Mobile phone apps capable of monitoring arrhythmias and heart rate (HR) are increasingly used for screening, diagnosis, and monitoring of HR and rhythm disorders such as atrial fibrillation (AF). These apps involve either the use of (1) photoplethysmographic recording or (2) a handheld external electrocardiographic recording device attached to the mobile phone or wristband.

**Objective:**

This review seeks to explore the current state of mobile phone apps in cardiac rhythmology while highlighting shortcomings for further research.

**Methods:**

We conducted a narrative review of the use of mobile phone devices by searching PubMed and EMBASE from their inception to October 2018. Potentially relevant papers were then compared against a checklist for relevance and reviewed independently for inclusion, with focus on 4 allocated topics of (1) mobile phone monitoring, (2) AF, (3) HR, and (4) HR variability (HRV).

**Results:**

The findings of this narrative review suggest that there is a role for mobile phone apps in the diagnosis, monitoring, and screening for arrhythmias and HR. Photoplethysmography and handheld electrocardiograph recorders are the 2 main techniques adopted in monitoring HR, HRV, and AF.

**Conclusions:**

A number of studies have demonstrated high accuracy of a number of different mobile devices for the detection of AF. However, further studies are warranted to validate their use for large scale AF screening.

## Introduction

Over the past years, there has been a significant increase in the number of mobile phone apps focusing on cardiovascular diseases. These apps are designed to monitor cardiovascular risk factors such as obesity, smoking, sedentary lifestyle, diabetes, and hypertension as well as to prevent and manage chronic conditions such as atrial fibrillation (AF) [1-5]. There are currently more than 100,000 available mobile health apps on iTunes and Google Play as well as more than 400 wearable activity monitors [6]. Approximately 64% of adults possess a mobile phone in the United States, and 62% of mobile phone owners use their phones to access health information and obtain education about diseases and health conditions [6]. Previous research has reported the benefits of using mobile health in modifying behavior to improve cardiovascular health through dietary management, physical activity promotion, smoking cessation, weight control, blood pressure control, cholesterol management, and blood sugar measurement [7-12]. However, there remains some ambiguity when it comes to the use of such apps in heart rate (HR) and HR variability (HRV) because of the lack of reliable statistical tests used in studies and the lack of standardized recommendations from professional bodies in terms of screening and monitoring of these parameters, as highlighted by Pecchia et al recently [13].

Currently, mobile phone–based photoplethysmography (PPG) and handheld electrocardiograph (ECG) recorders are the 2 main concepts adopted in monitoring HR, HRV, and AF [14,15]. PPG is an optical technique that detects heartbeats by analyzing changes in skin color and light absorption. Using a mobile app, the PPG sensor detects variations in light intensity via transmission through or reflection from the tissue (the reflectance and transmittance model). The variations in the light intensity are related to changes in the blood perfusion of the tissue, and based on these changes, heart-related information can be retrieved [14,16]. Similarly, the handheld ECG recorder is also based primarily on a mobile phone and mobile phone app interface. However, an additional external component, an ECG sensor unit, is required for the app to function adequately. This will then allow for a standard lead I ECG to be recorded [15]. For the purpose of this review, the term *handheld ECG recorder* will refer specifically to the mobile phone app involving an external ECG sensor component, which is not required by the PPG approach.

Studies have been conducted using mobile phone apps, such as AliveCor, to detect arrhythmias such as AF [17-19], atrial flutter, atrial and ventricular premature beats, bundle branch blocks, and ST-segment abnormalities [20]. As the use of mobile phone apps in cardiovascular care is a rapidly evolving field, this literature review seeks to act as a checkpoint, encapsulating the current state of mobile phone–based PPG and ECG recorders in monitoring HR, HRV, and AF. This will put into perspective what has been effective and applicable along with the relevant challenges encountered to better navigate future research projects into this niche.

## Methods

### Overview

A PubMed and EMBASE search that primarily concerned the use of mobile phone apps for monitoring HR, HRV, and AF was conducted. The following search string was employed: (“mobile applications” OR “smartphone” OR “digital health”) AND (“atrial fibrillation” OR “heart rate”). The search period was determined as beginning from the earliest available publications in the databases to October 14, 2018. No language restrictions were included.

The search results were subsequently transferred to Microsoft Excel (Microsoft Corp, Redmond WA), and all potentially relevant reports were retrieved as complete manuscripts and abstracts and assessed for compliance with relevance to the 4 core principles of this narrative review: (1) mobile phone apps, (2) HR, (3) HRV, and (4) AF. Overall, 3 authors (CL, FW, and TT) independently reviewed each publication and report, and findings were agreed upon by consensus with input from 2 senior researchers (BY and GT). A total of 1006 papers were found, and 822 publications were excluded after screening due to irrelevance and noncompliance with principles explored. Thus, 124 papers were used for this narrative review (Multimedia Appendix 1).

## Results

### Atrial Fibrillation and Other Arrhythmias

#### Atrial Fibrillation—KardiaMobile App

The KardiaMobile app, developed by AliveCor [21], is a commonly used device in studies investigating the monitoring of AF. The equipment comprises a finger pad sensor for the left and right hands, which is attached to the mobile phone, and an app that receives the transmitted information. A 2017 study by Tu et al gives a detailed account of the technology used by the device [22]. When fingers are placed on the sensors, recording begins. Using a 19,000 Hz center frequency and a modulation index of 200 Hz/mV, electrical activity is transmitted to the mobile phone via ultrasound signal frequency modulation to obtain the ECG trace. The frequency and modulation index signal that is transmitted to the microphone of the mobile phone are digitized at 44.1 kHz and 24-bit resolution, respectively. The app is then able to produce an ECG trace from the received signal (300 samples/second, 16-bit resolution), which can either be viewed as it is produced or be stored as a PDF file for later viewing. On the basis of the absence of P waves and irregularity of the RR (R wave to R wave) interval, the associated algorithm within the app produces a potential AF diagnosis [22].

The Lau et al study [23] took a group of 109 patients, 39 of which were in AF, and used the AliveCor algorithm to analyze the rhythms, obtaining a sensitivity of 87% and specificity of 97% when compared with interpretation by a cardiologist. The algorithm was then altered by increasing the weighting of absent P waves in the diagnostic process, and recordings were conducted on this same set of patients. The optimized algorithm was found to have a sensitivity of 100% and specificity of 96%. A second set of 204 patients, with 48 having known AF, were then analyzed by the optimized algorithm, which obtained a sensitivity of 98% and specificity of 97% in this second dataset. Other studies have largely found the sensitivity of the AliveCor device to be in the region of 90% to 98%, with specificity values ranging from 29.2% to 99% in various studies [24-29]. However, a study on the use of AliveCor in inpatients in cardiology and geriatric wards only found a sensitivity of 54.5% and 78.9%, respectively, for these groups [26], whereas another study published in 2017 used the AliveCor algorithm as a control and found a sensitivity of 71.4% for AliveCor [30]. A study of 13,222 patients identified 101 participants to have previously undiagnosed AF, with 65.3% of these patients reporting no symptoms before this diagnosis [24]. Within the field of pediatrics, accurate single-lead ECG tracings were also obtained in both healthy children and children with arrhythmic abnormalities [31]. The device is generally well tolerated, and patients report that it is easy to use [27]. Implementation of the AliveCor in community health care settings has also been investigated. Patients generally responded positively to the suggestion of an ECG being conducted by a pharmacist while they were attending the pharmacy for other purposes [32]. However, some patients were unwilling to have this conducted for the fear of discovering something was wrong. In a primary care setting, the use of AliveCor was well received by patients, but the staff had some reservations about being involved because of a self-perceived lack of knowledge [33]. Patients also report that they felt more confident in managing their AF by using the app [27,34]. The AliveCor device [18,35-37] has also been incorporated with patient education to increase the knowledge of the patients about AF [38]. Mobile apps have been extended to monitor paroxysmal AF for intermittent anticoagulation [39]. This evidently raises public health issues of systematic and opportunistic screening. In a recent evaluation of the European Heart Rhythm Association Consensus on screening AF, opportunistic screening was recommended for individuals aged above 65 years and younger patients at high risk of stroke [40]. As such, it is important to understand local screening guidelines before systematic or opportunistic screenings commence as large number of false positives yields unnecessary, expensive, and potentially harmful follow-ups that might affect patients’ mental health.

#### Atrial Fibrillation—CardiioRhythm App

The detection of AF by analysis of finger and facial PPG signals using the CardiioRhythm mobile phone app has been tested in 2 recent studies. In the study by Chan et al [30], finger PPG waveforms of 20-seconds duration were captured 3 times using an iPhone 4S equipped with the CardiioRhythm app and compared with the AliveCor single-lead ECG as referenced. Finger PPG waveforms were captured using the light emitting diode flash of the iPhone and the camera, which detected reflected light to measure the arterial pulsation. These waveforms were recorded at 30 Hz, with the duration of each recording being 17.1 seconds, and were filtered with a bandpass filter using a range of 0.7 Hz to 4.0 Hz. AF was diagnosed from this if 2 of the 3 PPG recordings were irregular. This was defined as a lack of repeating pattern, using a Support Vector Machine to classify the waveforms as similar or nonsimilar to other waveforms. Analysis of the diagnoses put forward by the CardiioRhythm app in this study showed a sensitivity of 92.9% and specificity of 97.7% in detecting AF compared with a sensitivity of 71.4% for the AliveCor device that was obtained by this study. This study found the specificities for the 2 devices to be comparable. The first prospective, international, 2-center, clinical validation study (DETECT AF PRO) based on similar finger PPG technology was also conducted recently to demonstrate the feasibility of such apps alone with a 5-min sensitivity and specificity of 91.5% and 99.6%, respectively [41].

**Figure 1:**
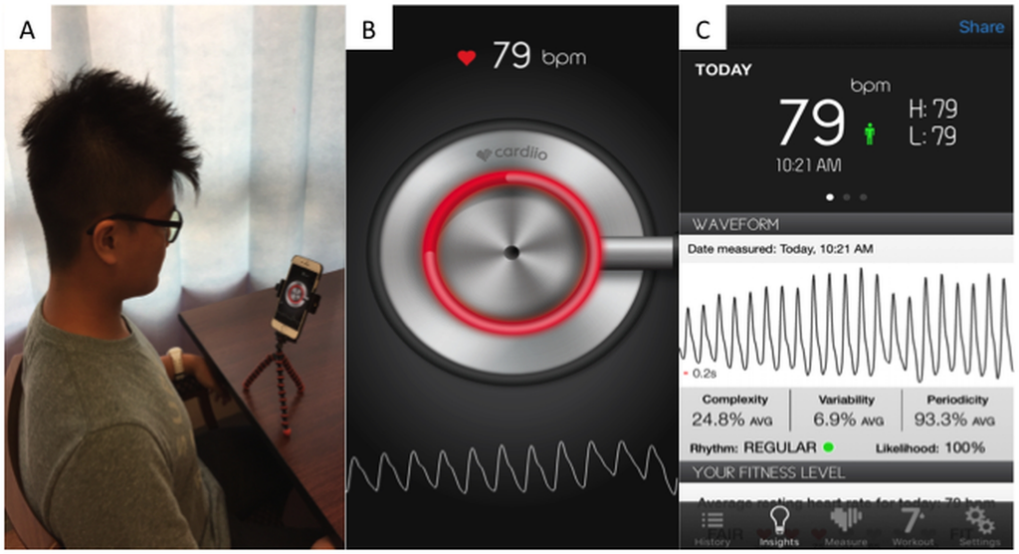
(A) Patient setup; (B) cardiioRhythm app facial photoplethysmography analysis interface; and (C) complete facial photoplethysmography signals report.

In another study by Yan et al, the CardiioRhythm app was used to analyze facial PPG signals in 217 patients recorded using the front camera of an iPhone 6S without physical contact (Figure 1) [42]. Overall, 3 successive 20-second recordings were acquired per patient. Pulse irregularity in 1 or more PPG readings or 3 uninterpretable PPG readings were considered a positive AF screening result. The CardiioRhythm facial PPG app demonstrated a sensitivity of 94.7% and specificity of 95.8%. The positive and negative predictive values were 92.2% and 97.1%, respectively.

#### Atrial Fibrillation—FibriCheck App

A recent study conducted by Mortelmans et al [43] used the PPG app FibriCheck. The patient places the left index finger over the flashlight and camera, holding their finger horizontally and keeping the finger in place for 1 min. To ensure an accurate reading, the screen becomes red when appropriate contact has been made, and the practitioner manually commences the measurement. The amount of light that is reflected onto the camera is then captured and used to calculate the variation in local arteriole blood volume pulse variation. The rhythm of the pulse is then identified based on the RR interval. The app in the mobile phone then judges the quality of the signal based on the detection of the pulse. If the detection was masked with noise, or beats were absent, these recordings were not included in the app’s analysis. When measurements were detected as a good signal, these data were interpreted by the AF algorithm.

Concurrently with the PPG measurement, a single-lead ECG was taken and analyzed by the FibriCheck app. Selection of the data took place based on quality, and good quality measurements were analyzed by the AF algorithm using variability between RR intervals to detect irregularities. The FibriCheck app was used on an iPhone 5S. With 242 participants, this study found a sensitivity of 98%, specificity of 88%, and accuracy of 93% for the FibriCheck algorithm when the data were obtained via PPG. Interpretation by the app of the single-lead ECG performed better, yielding a sensitivity of 98%, specificity of 90%, and accuracy of 94% when compared with standard ECG. When comparing the 2 methods by false-positive results, when analyzed by FibriCheck, 8 false positives were produced from a trace obtained by PPG measurement and 11 false positives were produced from a trace obtained by a single-lead ECG.

#### Atrial Fibrillation—Other Methods of Detection

Some studies have looked at the use of the inertial measurement unit, using modern microelectromechanical (MEMS) sensors already present in mobile phones, to detect AF [44]. The patient is advised to lie supine and place the mobile phone on their chest. The accelerometer of the mobile phone, which detects the orientation of the phone, can detect cardiogenic movements of the chest. The movements detected are those caused by the opening of the aortic valve, and so, the interval between each successive valve opening is recorded and considered to determine the presence of AF. This yielded a sensitivity of 93.8% and specificity of 100% in the detection of AF. A similar study used the MEMS sensors of mobile phones to obtain measurements, which when analyzed by machine learning methods found a sensitivity of 98.5% and specificity of 95.2% in its best performing method [45].

The feasibility of producing a wearable sensor that records HR and transmits information to a mobile phone app was examined [46]. The way this app identifies AF is similar to the FibriCheck app. As an example, a 42-year-old male admitted with new-onset AF of undetermined duration was deemed appropriate for electrocardioversion because of his FitBit Charge HR device recording the time at which increased HR was observed, allowing the onset of AF to be identified [47]. In a study by Yan et al, the diagnostic accuracy of the CardiioDeepRhythm app, a deep convolutional neural network for detecting AF from the PPG signal acquired using an off-the-shelf wrist-worn device (Empatica E4, Milan, Italy), was tested in 51 in-hospital patients reporting a sensitivity and specificity of 93% and 94%, respectively [48]. These findings demonstrate the promising value of PPG sensors for ambulatory AF monitoring. Krivoshei et al [49] conducted a study investigating the use of PPG in AF, with a view to implementing the technology in a smartwatch; the protocol for this trial was published as an abstract during a conference [50]. Table 1 summarizes the sensitivities and specificities of the apps in AF detection. Despite some studies conducting direct comparisons between different technological modalities [30], it is still done with reference to the gold standard of a conventional 12-lead ECG within a short period. This is especially important because none of the new app technologies have been properly integrated into the wider health care network as compared with the 12-lead ECG, which is a validated instrument commonly available within primary and secondary care settings.

**Table 1:** Sensitivities and specificities for the 5 main technologies applied in atrial fibrillation detection.

Modalities	Sensitivity (%)	Specificity (%)
Original AliveCor algorithm (finger pad sensor)	54.5-98.0	29.2-99.0
Optimized AliveCor algorithm (finger pad sensor)	98-100	96-97
CardiioRhythm (finger PPG^a^)	92.6-92.9	94.8-97.7
CardiioRhythm (facial PPG)	94.7	95.8
CardiioRhythm (wristband PPG)	93	94
FibriCheck (finger PPG)	98	88-90
Inertial measurement unit (chest accelerometer)	93.8-98.5	95.2-100

^a^PPG: photoplethysmography.

### Heart Rate and Heart Rate Variability

#### Heart Rate

There has been considerable innovation in the use of mobile phone apps for the purpose of HR monitoring in adults. In particular, numerous studies have investigated the potential of 2 specific methods—seismocardiogram (SCG) and PPG—in producing accurate HR measurements. Landreani et al, for instance, described the application of SCG and ballistocardiogram (BCG) signals for HR monitoring apps in mobile phone–embedded accelerometers [51]. This is achieved by determining the RR interval of sufficient amplitude and subsequently detecting the fiducial peak from the SCG and BCG signals. In PPG, the accuracy of this method was also demonstrated in a study by Alaleef et al, who found a 99.7% accuracy and maximum absolute error of 0.4 beats/min [52]. The accuracy and feasibility of PPG signals from mobile phone apps also varied between different types of PPG apps. In Parpinel et al’s study, higher feasibilities and accuracies were found for contact PPG–based apps compared with the noncontact PPG–based ones [53]. Koenig et al [54] found that their algorithm using PPG to assess HR had a consistent accuracy when compared with ECG (correlation index R>.99).

##### Gold Standards and Validation of Mobile Phone Heart Rate Apps

Although there is currently no consensus on the gold standard for the validation of HR apps, Vanderberk et al suggested the comparison of the HR on mobile phone apps with an ECG system via RR intervals. With regard to the actual accuracy of these mobile phone apps, there was no significant difference (*P*=.92) found between the interval measurements of the HR app and the ECG system, suggesting that the HR apps have a comparable accuracy with ECGs [55]. This is further supported by a recent comparison of 3 HR apps against simultaneous standard ECG monitoring, which found an acceptable correlation. However, accuracy became questionable in specific cases of irregular rhythms such as AF [56].

##### Heart Rate Apps in Different Fields

The use of HR apps has been tested in different clinical settings. In a specific app reviewed by Chaudhry et al, named *Unique Heart Rate Monitor*, PPG was used for the measurement and categorization of workout intensity. It also allows subjects to check their HR in response to medical therapy [57]. The clinical use of HR apps during exercise was also assessed by numerous studies. In particular, some studies noted that the measurement accuracy tended to differ at different exercise intensities. In 1 study, the app had higher measurement errors at increased exercise intensities when compared with Holter echocardiography monitors; however, it had similar accuracy to the Holter monitor at resting and recovery stages [58]. Yan et al presented similar findings, although the correlations between the facial PPG–estimated HR and ECG HR had smaller variations (*r*=.997 on resting compared with *r*=.982 with postmoderate intensity exercise) [59]. Furthermore, it was also found that both iOS and Android operating systems, with the same HR app, had concurrent validity with an FT7 Polar HR monitor at rest and at postexercise time points [60]. Although many studies reported the use of mobile phones as the media for measuring and interpreting HRs, some studies used mobile phone apps as a feedback system rather than the actual HR sensor. By using Bluetooth communication, subjects had their HR measured by a specially devised HR sensor at different exercise intensities, and this information was transferred via Bluetooth back to the mobile phone for analysis based on existing information during the pre-exercise period [61]. In addition, some cardiac HR monitors have a built-in alert system with real-time bradycardia and tachycardia arrhythmia detection. In the study by Golzar et al, users reported almost zero delays with data transmission and a 91.62% performance accuracy in comparison with regular ECG monitoring [62].

Apart from using HR apps for exercise HR monitoring, other studies also investigated the potential use of HR monitoring to reduce the exercise-induced risk of hypoglycemia. This is done within the context of control-to-range closed-loop artificial pancreas systems. By integrating HR as part of the feedback system in the Android app, it was found that the inclusion of HR monitoring better controlled blood glucose decline during exercise (*P*=.02) and resulted in fewer hypoglycemic events during exercise (*P*=.16 for none vs 2 events) [63].

The use of camera-based PPG has also been suggested for use in other age groups, including infants and newborns. Although the method has reported inaccuracies because of subject movements throughout the day, Kevat et al found that this method of HR measurement had increased clarity and precision in neonates receiving phototherapy [64]. Although this specific application of PPG may have potential use in pediatric patients, the inaccuracies of this method remain. Thus, when PPG is compared with normal ECG monitoring, different phone apps had lower accuracies for HR measurements using the finger or toe but higher accuracies at the earlobes. HR measurements were inaccurate above 120 bpm [65]. These results suggest that the technique is still evolving and requires considerable research before implementing it as a standard alternative to regular ECG HR monitoring for pediatric populations.

Apart from HR measurements in neonates and children, Android-based HR apps have also been developed to track and increase adherence to certain activities such as breathing awareness meditation. In the study by Gregoski et al, a tension tamer HR app used PPG via phone camera lens to transmit time-stamped HRs back to the hospital server for real-time adherence collection [66]. However, the very small sample size prevents definite conclusions to be drawn. The inaccuracies of these mobile phone apps were also found in the context of fetal HR monitoring by Soffer et al, who reviewed 30 unique apps and found that over 33% of the apps did not put disclaimers and/or provided false medical information [67].

Although PPG has been widely investigated, the use of mobile phones to generate phonocardiograms (PCGs) has been reviewed by many investigators such as Chen et al, who used iPhone 4S to record heart sounds from subjects at rest or postexercise and used these sounds for HR calculation in PCGs via the peak-based detection method [68]. Although the use of this Web-based PCG-template extraction and matching was found to be accurate, further research needs to be done to validate this finding.

#### Heart Rate Variability

Apart from HR, HRV measurements in mobile phone apps have also been extensively researched. Sometimes coined as the *RR interval*, HRV is the measure of the variation in time intervals between heartbeats, usually with reference to ventricular contraction. Classically, HRV is measured using the ECG using mainly the time-domain and frequency-domain approaches introduced in 1996 [69,70].

With the use of PPG, Bolkovsky et al used both Android and iPhone mobile phones to obtain RR intervals and subsequently deduce HRV via complex HRV algorithms. Although the results were statistically same as the ECG gold standard, the main issue was the insufficient sampling rate in both phones (20 Hz in Android and 30 Hz in iPhone), which was below the suggested rate of 250 Hz [71]. mobile phone PPG has also been advocated by Plews et al, who reported almost perfect correlations of PPG with the ECGs (R=.99), with an acceptable technical error of estimates and trivial differences in standardized differences [72]. The accuracy and reliability of mobile phone PPGs have also been validated in other studies; in some cases, mobile phone PPGs were found to have comparable accuracies with commonly used HRV computer software programs (R=.92) [73]. Other methods of HRV measurement have also been studied, such as seismocardiography. Although sampling frequency from mobile phone devices accounted for a significant source of error, RR series measurements differed by less than 10 ms in some studies, suggesting the comparable accuracy of this measuring technique [74].

Although many studies support mobile phone PPG, common errors in using this low-cost technology include frequent noise and artifacts in measurement. To reduce the impact of noise, however, Huang et al proposed the use of a continuous wavelet transform denoising technique to extract the pulse signal and subsequently deduce the RR intervals in the denoised signal. The experiments showed low mean absolute errors of only 3.53 ms, highlighting the efficacy of this proposed method in reducing mobile phone PPG errors. The use of this technique for error reduction should thus be investigated further [75].

Although methods have been proposed to reduce the impact of noise and artifacts in HRV measurement, other studies have investigated the effect of mobile phone models on the SD of beat-to-beat error measurement (SDE) of HRV indices. In 1 study, 2 different mobile phone models (Samsung S5 and Motorola) showed significant device influence in the supine posture measurement, with the Motorola model having a higher SDE than the Samsung S5 [76].

##### Application of Heart Rate Variability

In 1 case report, Lai et al reported the potential use of HRV measurement analysis in monitoring concussed athletes as well as assessing their capacity to *return-to-play* [77]. Via an *AliveCor mobile phone ECG* app, HRV parameters were statistically significant in symptomatic and recovered concussed athletes. However, the study noted that this difference was affected by the severity of traumatic brain injury, whereas other similar studies reported no difference in HRV parameters [78,79]. Other studies also proposed the potential use of mobile phone–derived HRV in athletic training programs; for instance, collection of daily HRV data on mobile phones using ultrashort HRV measures provides trainers with numerical indicators on athlete coping and adaptation [80]. Similar studies by Flatt et al also found that measuring the log-transformed root mean square of successive RR intervals (RMSSD) multiplied by 20 (lnRMSSDx20) obtained by mobile phone apps were sensitive markers to the changes in training load in soccer team’s training program [81].

HRV by mobile phone apps have also been used as an accurate predictor of acute mountain sickness (AMS) at high altitudes. In 1 study, Mellor et al reported lower HRV scores in those with mild and severe AMS compared with those without AMS (*P*=.007) and found that a reduction in HRV greater than 5 had an 83% sensitivity and 60% specificity of identifying severe AMS. As this is the first study of its kind, further studies should be conducted to confirm these findings [82].

HRV measurement by mobile phone apps was also investigated with regard to the autonomic nervous system and mental health. For instance, Heathers et al found that pulse rate variability (PRV) could be measured by mobile phone substitutes with accuracies ranging from 2% to 5% for low- and high-frequency spectral power, respectively [83]. Mobile phone apps, thus, may play a future role in psychophysiological research. In addition, mobile phones have also been used in stress classification via the use of night-time HRV data and as a monitoring tool for mental stress in different psychological settings [84,85]. The drawbacks of these studies, however, are the fairly low accuracies (59%) and that the HRV measurements were confined to ultra-short periods. The potential for long-term measurement should thus be investigated [85].

### Other Arrhythmias

Although a large proportion of the research has investigated the use of mobile apps to detect AF, other arrhythmias have been investigated. A study presented in abstract in 2016 by Dimarco et al [86] used the AliveCor device to investigate 148 patients who had reported palpitations for arrhythmias. The device was used as an alternative to ambulatory monitoring.

An ECG trace was obtained using the device, and the trace was then interpreted by a cardiologist. The traces obtained were of suitable quality to diagnose arrhythmias, with diagnoses of supraventricular or ventricular ectopy in 27.4% of patients, sinus tachycardia in 18.6%, AF in 7.1%, and supraventricular tachycardia in 5.3%, which could be managed appropriately. In the remaining 41.6%, they could be reassured that they remained in sinus rhythm.

When the AliveCor device was used to measure corrected QT (Q wave to T wave) interval in those with sinus rhythm and antiarrhythmic drugs, results suggested that it was comparable with 12-lead ECG [87]. With a sensitivity of 64% and specificity of 97%, this suggested that the device could be used to monitor those with arrhythmias. A study comparing the AliveCor device with a 14-day event monitor found that the device was equivalent to the event monitor in detecting AF and premature atrial contractions (PAC) but was slightly less effective in identifying episodes of premature ventricular contractions (PVC) [88].

The Smartphone Pediatric ElectrocARdiogram trial [89] investigated the use of the AliveCor device in pediatric patients with known paroxysmal arrhythmias. The device produced a trace, which was then reviewed by a cardiologist. Of the 240 traces recorded by 20 patients, 231 were suitable for diagnostic purposes. A total of 35 patients were initially included in the trial; 15 of these patients did not transmit ECGs. Of the suitable traces, sinus rhythm was detected in 43% of the traces. Moreover, 29% displayed sinus tachycardia, supraventricular tachycardia was present in 16%, and AF was present in 8%. Furthermore, 98% of parents of patients involved in the trial reported greater comfort in managing their child’s arrhythmia, with 93% expressing a desire to continue using the device.

A study by Mc et al [90] assessed whether the Pulse Waveform Analysis app could distinguish sinus rhythm and AF from PAC and PVC. The patient covered the camera and lamp of an iPhone 4S with their finger for 2 min, allowing a pulse waveform to be recorded and a video of blood flow to be recorded. A total of 3 statistical techniques were then used in combination—RMSSD, Poincare plot, and Shannon Entropy (ShE)—to distinguish AF from ectopic beats. Results for each of the arrhythmias were as follows: for AF, a sensitivity of 97%, specificity of 93.5%, and accuracy of 95.1%; for PAC, a sensitivity of 66.7%, specificity of 98%, and accuracy of 95.5%; and for PVC, a sensitivity of 73.3%, specificity of 97.6%, and accuracy of 96%. A similar study assessed the use of a mobile phone app identifying PVC using RR intervals, finding a sensitivity of 90.13% and specificity of 82.52% [91]. A study using similar methods found that the best results were obtained when RMSSD and ShE were combined to analyze the trace. When these 2 techniques were combined in a study of 76 participants, the results were 100% accurate for detecting AF and 96.05% accurate for detecting sinus rhythm [92]. Using simulated ECG signals from the Massachusetts Institute of Technology–Beth Israel Hospital arrhythmia database, a study tested the ability of an Android program to detect arrhythmias from ECG traces [93]. The analog ECG traces were converted to a digital format and then made available for transmission via Bluetooth. The Pan-Tompkins algorithm [94] was then used to identify RR intervals to determine the heartbeat. The algorithm produced an accuracy of 98.98% at detecting sinus rhythm and an average accuracy of 98.34% at detecting 7 different possible heart rhythms.

## Discussion

### Limitations

The main limitations of this narrative review are the lack of focus on more specific arrhythmias beyond normal sinus rhythm and AF. However, normal sinus rhythm and AF are epidemiologically the most common types of heart rhythm; hence, it is important to establish a strong foundation for these apps with these rhythms first. Second, it is common for narrative reviews to circumvent important criteria to mitigate bias because of the lack of a rigorous evidence-based methodology. This inevitably leads to selection bias based on expert opinions. This bias was addressed in this narrative review by adapting the Preferred Reporting Items for Systematic Reviews and Meta-Analyses criteria to select relevant publications. In doing so, all evidence pertaining to the issues discussed in this review has been considered to provide a balanced understanding and discussion.

#### Photoplethysmography

Despite being effective in accessing HR and HRV, the applications of PPG monitoring are limited by multiple confounders such as finger pressure, skin tone, light intensities, and user movement leading to artefactual measurements [95,96]. As such, this will have an impact on the feasibility and reliability of mobile phone–based PPG within clinical practice. A minimum sampling rate is necessary for clinically accurate measurements—30 Hz for HR and 200 Hz for HRV measurements [97,98]. However, the frame rate of mobile phones usually operates around 30 Hz, which is a major limitation identified by Bolkhovsky et al [71]. It was proposed that using cubic interpolation, the second derivative, and the zero-crossing algorithm instead of minima detection would overcome this limitation and allow for better HR detection [99-102]. A filter is required to remove artifacts without compromising the original signal when conducting a time-domain analysis to evaluate small variations occurring in normal-to-normal intervals [103]. Examples of filters are independent component analysis using accelerometer data to remove artifacts [104] or employing a fourth-order bandpass filter [99,101]. A heating problem was also noticed by Garcia-Agundez et al when testing their algorithm, which resulted in complaints about the discomfort of holding the mobile phone [102]. Furthermore, although the SCG accelerometers could be used without supporting ECG signals, HR detection was only possible if patients were motionless and supine, leading to considerable variability in data collection because of different measurement positions. There were also difficulties encountered in setting appropriate R-peak thresholds and in comparing the data with simultaneous ECG signals (the gold standard technique) [105,106]. Another area to consider is the use of PP (P wave to P wave) intervals and PRV instead of the RR interval and HRV. Although most studies use RR and HRV approaches with supporting literature that time domain, frequency domain, and Poincare plot HRV parameters computed using RR interval and PP interval methods showed no significant differences, the PP variability was found to be accurate (0.1 ms) compared with RR variability [107]. Furthermore, most studies usually compare between normal sinus rhythm and AF. However, a simple ectopic will be able to derange specificity to unacceptable levels. In this respect, more research needs to done to evaluate the impact of short-term arrhythmic abnormalities on PPG-based and single-lead ECG–based AF detection modalities.

#### Handheld Electrocardiograph Recorders

Handheld single-lead ECG recorders, which correspond to the standard lead I, do not cater to potential positional variability. For example, in cases where the heart is positioned more vertically, the amplitude of the QRS complexes may be reduced and comparable with the amplitude of artifacts [15]. Furthermore, a single recorded lead I ECG also does not allow for differentiation between types of narrow and wide complex QRS tachycardia [15]. Although small studies have been conducted to show that it is plausible to use a single-lead ECG to diagnose the ST-segment elevation acute coronary syndrome [108], it is not sufficient to warrant routine clinical use. In some situations, new materials and sensors (eg, biopatches) have allowed these ECGs to be recorded from atypical places (eg, mastoid area) [109] and under different environmental conditions such as after immersion in water or in areas with magnetic fields between 1.5 and 3 T (during magnetic resonance imaging) [110-113]. Novel biopatches have also allowed other parameters such as respiratory rate, body position, temperature, and quality of sleep or physical activity to be monitored, which aids in excluding ST depression caused by increased physical activity or changes in body position [19,114,115]. However, when this is coupled with fewer leads, not only will there be suboptimal signal-to-noise ratio but signals generated might also be different from those produced by standard ECG leads [15]. Furthermore, the utility of biopatches comes with a drawback of having a short intersensor distance, which could potentially compromise the quality of the P wave recorded [15].

#### Health Care Infrastructural Implementation

Despite being a promising concept, there are currently clear limitations to the use of mobile phone apps for HR, HRV, and AF monitoring. Looking beyond the technological drawbacks, there is little evidence as to how mobile phone monitoring will be matched with health care infrastructural changes to allow for such data to sync with the electronic medical record [116,117]. Furthermore, not all patients will have the same device or mobile phone, which may result in different recorded results. Special clinical attention also has to be given to incidental findings of ECG abnormalities such as ventricular and supraventricular tachycardia in asymptomatic low-risk patients [15]. The impact of app use on health care services is also not to be underestimated. The necessity for a health care professional to confirm arrhythmias detected by these apps will result in a significant burden on services. Given the transient nature of some arrhythmias, an app with a high specificity is likely to be more beneficial to avoid over investigation and treatment of patients, reducing the impact on existing services.

A recent large-scale community AF screening program on more than 10,000 patients aged above of 65 years was conducted, proving the feasibility of integrating this technological within-health care services to identify with new AF [118]. The main drawback illustrated by this study is the lack of downstream management pathways. This raises the medico-legal aspect of introducing mobile phone apps. First, there must be an understanding as to how these apps will *support* decision making or purport to intervene in clinical decisions. This, in turn, will lay the foundation for a robust government framework to evaluate its effect on clinical outcomes and potential unintended consequences [119,120]. A deeper engagement in this respect was raised in January 2016 when 2 class action lawsuits were filed against such manufacturers, calling into question the reliability and accuracy of these devices and apps [121]. Perhaps the issue is in classifying these devices and apps as certified *medical devices*. Despite the intuitive need for medical devices to be of high quality, local regulations for *medical devices* have to be followed. Such regulations range from the Medical Devices Framework concept of *intended purpose* to the risk-based case-by-case approach employed by the US Food and Drug Administration [122].

Looking specifically at the European regulations, the Medical Device Directive was replaced by the new Medical Device Regulation. Although the new regulatory framework still revolves around *intended purpose*, the bar for *medical device* classification has been lowered because of a broader definition assigned to the term *medical purpose*. In short, despite the undisputed potential of such apps in the detection of arrhythmias, adhering to appropriate laws and regulations remains a significant hurdle to address [123].

### Conclusions and Future Directions

In this study, we conducted a narrative review of the literature surrounding the usage of mobile phone apps in the monitoring of HR and rhythm. The findings of this narrative review suggest that there is a role for mobile phone app in the diagnosis, monitoring, and screening of arrhythmias and HR. The usage of apps in specific situations, such as during and following exercise or to measure corrected QT interval following the administration of medications, has also shown a role for the apps in more specific scenarios. Although the majority of literature reviewed focused on adult patients, the use of PPG apps in pediatric and neonatal patient populations requires further studies.

Some problems identified with the use of these devices have included patient movement resulting in artifact and in positional variability in patient usage affecting results. This has been demonstrated in studies investigating both adult and pediatric patients [89]. There is also an issue regarding consistency and availability of mobile phones, with variation in the devices used. Within the context of HR monitoring and AF detection, given the impressive degree of sensitivity (>90%) and specificity (>90%) in most cases or apps, neither sensitivity nor specificity is more important than the other. Instead, it is important for both sensitivity and specificity to be maintained at a high level or be on par with the standard ECG, especially in patients with pacemakers or implantable cardioverter defibrillators [124]. Therefore, further studies are required to address these issues before they can be routinely implemented. As such, there are little implications for current practice at this point.

Moving forward, further large-scale validation studies looking beyond normal sinus rhythm and AF are required. For example, it is important for these apps to not mistake malignant ventricular ectopics as sinus rhythm or AF as it will be potentially harmful to patients. With regard to AF detection, multiple studies have been conducted to validate its feasibility. Perhaps it would be best to start conducting large-scale AF screening programs. Doing so will help address the following points: (1) the medico-legal aspect of implementing these apps as valid *medical devices* for systematic and opportunistic screening nationally, (2) the cost-effectiveness of identifying new AF patients through such a screening program, and (3) refinement of a suitable management pathway for new AF patients identified through this process. Such an initiative has the potential to reduce AF burden internationally and increase the efficiency of picking up AF in the community. Within the clinical setting, apps used by patients can prompt clinicians to perform further investigations such as a confirmatory ECG.

## Appendix

Multimedia Appendix 1 A description outlining the search process of papers.

## Abbreviations

AF: atrial fibrillation
AMS: acute mountain sickness
BCG: ballistocardiogram
ECG: electrocardiograph
HR: heart rate
HRV: heart rate variability
MEMS: microelectromechanical sensors
PAC: premature atrial contractions
PCG: phonocardiogram
PP: P wave to P wave
PPG: photoplethysmography
PRV: pulse rate variability
PVC: premature ventricular contractions
QT: Q wave to T wave
RMSSD: Root Mean Square of Successive Difference
RR: R wave to R wave
SCG: seismocardiogram
SDE: SD of beat-to-beat error measurement
ShE: Shannon Entropy
