# Supplementary material for: The Current State of Mobile Phone Apps for Monitoring Heart Rate, Heart Rate Variability, and Atrial Fibrillation: Narrative Review
Source: JMIR Mhealth Uhealth. 2019 Feb 15;7(2):e11606. doi: 10.2196/11606 (PMC6396075; doi:10.2196/11606)
Supplement: Multimedia Appendix 1 [file mhealth_v7i2e11606_app1.pdf]

Multimedia Appendix 1. A flowchart outlining the search process of papers.

PubMed -

(‘mobile applications’ OR ‘smartphone’ OR ‘digital health’) AND (‘atrial fibrillation’ OR ‘heart rate’) – Total results (669)

Embase

1: mobile application/

2: smartphone/

3: digital health.mp.

4: 1 OR 2 OR 3

5: atrial fibrillation/

6: heart rate/

7: 5 OR 6

8: 4 AND 7 – (total results: 337)
